# From Deutsche Zeitschrift to International Journal of Legal Medicine—100 years of legal medicine through the lens of journal articles: Part 3: *Zeitschrift für Rechtsmedizin—Journal of Legal Medicine* from 1970 to 1990

**DOI:** 10.1007/s00414-023-02949-8

**Published:** 2023-01-28

**Authors:** Andreas Schmeling, Tony Fracasso, Heidi Pfeiffer, Ingo Wirth

**Affiliations:** 1 Institute of Legal Medicine, University Hospital Münster, Münster, Germany; 2 University Center of Legal Medicine (CURML), Geneva University Hospital, Geneva, Switzerland; 3 Berlin, Germany

**Keywords:** History of legal medicine, Official publications, Academic articles

## Abstract

From volume 67 (1970) onwards, the journal appeared under the new bilingual title *Zeitschrift für Rechtsmedizin—Journal of Legal Medicine*. The editorial board was expanded and internationalised. From 1970 to 1990, 1416 articles were published in 36 volumes. 1036 articles were in German and 380 in English. The authors of 411 articles came from non-German-speaking countries. Compared to the periods under review in the first two parts of our article series, the proportion of papers on forensic genetics increased significantly between 1970 and 1990, with a small increase in publications on the identification of unknown dead bodies. An opposite trend was observed in the articles on forensic psychiatry and psychology, sexual medicine and social medicine. This development reflects a further sharpening of the discipline’s profile.

## Introduction

On the occasion of the publication of the 100th volume of the *Deutsche Zeitschrift für die gesamte gerichtliche Medizin*, which continued under a new title, Gerchow [5] wrote in an editorial in 1988: “The first volume (1922) already showed the structure that was retained until 1969: congress reports, original papers and a section of abstracts. In the mid-1960s, it became apparent that this form of publication could not be carried on, especially as the section of abstracts was taking up more and more space.” As a logical consequence, the abstract section was segregated and continued as a separate journal with the title *Zentralblatt für die gesamte Rechtsmedizin und ihre Grenzgebiete*. From volume 67 (1970) onwards, the former *Deutsche Zeitschrift für die gesamte gerichtliche Medizin* was published under the new title *Zeitschrift für Rechtsmedizin—Journal of Legal Medicine*. The change of title had become necessary because the German Society for Forensic and Social Medicine, whose publication organ the journal was, had changed its name to German Society for Legal Medicine in 1968 [16]. The new bilingual title also showed the internationalisation of the journal. In addition to the title of the articles, the abstracts and the keywords preceding the article were now also published in German and English. Efforts to internationalise the journal were also reflected in the newly formed editorial board. This board included representatives from the German-speaking countries, Belgium, Denmark, Finland, France, Japan, Yugoslavia, Sweden, the USSR, UK, Hungary and the USA. The purpose of increasing the number of editors was to allow for double reviews of each manuscript. Another new feature was the introduction of categories. The categories covered editorials, reviews, original papers, case reports, obituaries and letters to the editor.

**Fig. 1:**
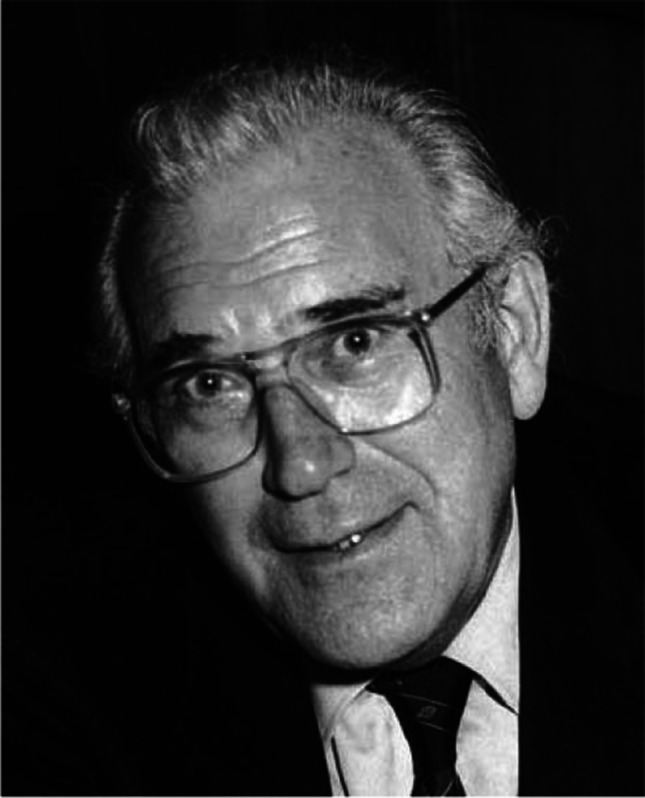
Joachim Gerchow, editor-in-chief of the *Zeitschrift für Rechtsmedizin—Journal of Legal Medicine* from 1970 to 1990 (photo reproduction courtesy of Hogrefe Verlag)

Throughout the period under review, Joachim Gerchow (Fig. 1) was editor-in-chief. For volumes 102 and 103, Bernd Brinkmann acted as second editor-in-chief, who then became the sole editor-in-chief from volume 104 onwards, when the journal was renamed *International Journal of Legal Medicine.*

In the first two parts of our article series “From Deutsche Zeitschrift to International Journal of Legal Medicine—100 years of legal medicine through the lens of journal articles”, the scientific publications appearing in the *Deutsche Zeitschrift für die gesamte gerichtliche Medizin* between 1922 and 1969 were reviewed [21, 22]. This analysis is now continued in the third part of the series for the articles published in the *Zeitschrift für Rechtsmedizin*—*Journal of Legal Medicine* between 1970 and 1990.

## Analysis of scientific articles

### General

**Fig. 2:**
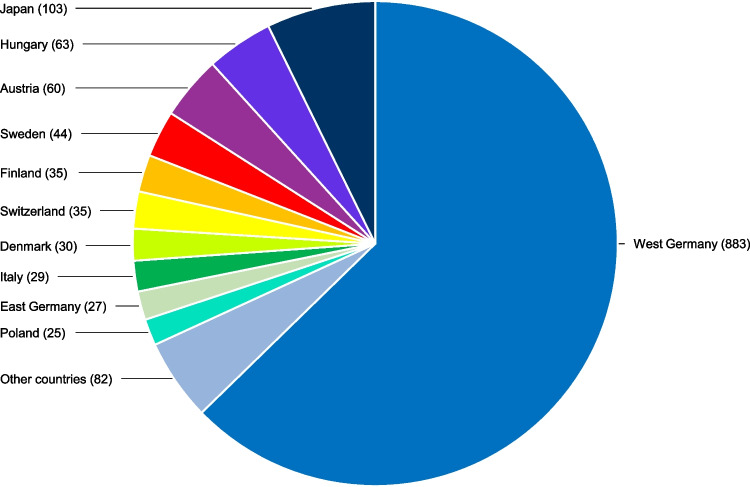
Countries of origin of the authors. The country of the first author determined the country of origin. The numbers in brackets indicate the number of articles written by authors from the respective countries. Other countries are USA (17), Spain (13), Belgium (12), CSSR (9), Bulgaria (7), Norway (7), Yugoslavia (3), USSR (3), UK (3) and Qatar (2). Brazil, China, France, India, the Netherlands and Thailand are represented with one article each

From 1970 to 1990, 1416 articles were published in 36 volumes. In terms of category, the articles included 3 editorials, 72 reviews, 990 original papers, 323 case reports, 10 obituaries and 18 letters to the editor. 1036 articles were in German and 380 in English. Figure 2 shows the authors’ countries of origin. The authors of 411 articles came from non-German-speaking countries.

The structure of the topics and their respective subtopics in the following content analysis of the scientific articles published in the *Zeitschrift für Rechtsmedizin*—*Journal of Legal Medicine* is the same as in Part 1 of our series of articles [21]. Table 1 provides an overview of the number of publications on the individual topics in the period under review.

**Table 1:** Overview of the number of publications on the individual topics in the period 1970 to 1990

Topic	Number of publications
History and development of the discipline	
History of the discipline	6
Development of the discipline	3
Personalia	10
Legal issues	31
Expert activities	21
General legal medicine	
External post-mortem examination/autopsy	71
Thanatology	68
Vital reactions	65
Forensic traumatology and pathology	
Forensic traumatology	209
Forensic obstetrics	8
Abortion	2
Filicide	1
Medical malpractice	39
Diseases and sudden natural death	135
Forensic toxicology	
Clinical signs of poisoning	67
Pathological anatomical findings	24
Toxicological chemical analyses	148
Alcohology	38
Identification of unknown decedents	38
Forensic genetics	
Blood group science	147
Forensic trace analysis	125
Parentage testing	53
DNA analysis	8
Scientific-technical criminalistics	24
Clinical legal medicine	1
Forensic psychiatry and psychology	9
Sexual medicine	1
Traffic medicine	44
Social medicine	2
Criminology	18
Total	1416

### History and development of the discipline

Six articles were published on the *history of the discipline*. Two papers deal with the historical development of the assessment of medical malpractice (86/303)^1^ and the estimation of the time of death (95/19). Another paper was dedicated to Paolo Zacchia, the “father of forensic medicine”, on the occasion of his 400th birthday (94/159). Three editorials gave a brief historical outline of the *Zeitschrift für Rechtsmedizin*—*Journal of Legal Medicine*.

In addition, there are 3 papers on the conception and *development of the discipline*. Berg (92/247) outlines the tasks of ethics committees at medical faculties and describes the contribution of legal medicine as follows: “It seems obvious to me that legal medicine has an important, not to say a decisive role to play in the ethics committee”. Compared to the pure lawyer, the medicolegal expert not only has the advantage of possessing a specialised knowledge of medical law but also of being familiar with hospital operations in contact with the patient due to his or her own basic medical training. Janssen (100/5) analyses the significance of morphological examinations for the administration of justice. He criticises the lack of binding guidelines for assessments with morphological content. In addition, he demands that at least a basic histological status, i.e. the examination of the large parenchymatous organs, must be carried out in every autopsy. In his article entitled “Forensic Medicine and Criminalistics” (102/421), Schwerd defines “medical criminalistics” as a small but important component of criminalistics. In doing so, he emphasises the extraordinary importance to be attributed to the inspection of the site where a corpse has been found by the medicolegal expert. Schwerd also criticises the term “forensic pathology”.

### Personalia

The tradition of honouring distinguished personalities of the discipline with laudations or commemorative publications was not continued during the period under review. However, 10 obituaries were published, namely for Friedrich Pietrusky (70/I), Fritz Schwarz (70/II), Jan Stanislaw Kobiela (71/249),^2^ Georg Strassmann (74/159), Kurt Walcher (74/321), Wilhelm Hallermann (77/321), Berthold Mueller (78/253), Milton Helpern (80/170), Unto Uotila (80/253) and Walter Krauland (102/77).

### Legal issues, expert activities

A review paper on recent developments in medical law dealt with legal issues of the diagnosis of brain death, embryo protection, (failed) sterilization, data protection and medical liability (91/165). Other publications on medical law concerned HIV tests without the knowledge of the affected person, assisted suicide and liability issues in the context of various medical complications as well as in the case of false expert reports (84/239, 86/21, 86/281, 98/245, 99/169). Althoff and Solbach analysed the outcome of criminal investigations against physicians (93/273). Issues of insurance medicine were discussed in three articles (75/299, 77/1, 77/237). Moreover, the legal basis of organ transplantation (78/215), case law on the practice of medicine pursuant to the Heilpraktikergesetz (Alternative Medical Practitioners Act) in Germany and euthanasia legislation in Sweden (70/32) were discussed.

Articles on *expert evidence* concerned the assessment of medical malpractice (71/67), causality issues (67/67, 75/61, 81/243), the classification of the manner of death (72/240, 77/313) and the cause of death (78/321) and the assessment of criminal responsibility (97/69). One publication deals with expert opinions on errors of assessment (101/49). Sources of assessment errors were deficiencies in the doctor’s expertise and negligent literature study, as well as illogical and unmotivated causal conclusions.

### General legal medicine

The *external and internal post-mortem examination* is dealt with in 2 papers on autopsy techniques. One addresses the dissection of the throat (88/257), the other describes how to dissect the larynx (96/11). Publications on complementary investigations in addition to the autopsy include deep freezing for intrathoracic organ fixation (67/218), coronarometry (75/241, 78/137), histological staining methods (90/269, 91/75) and microbiological testing (94/81, 96/297). In a review paper, Böhm describes the use of electron microscopy in legal medicine (92/77). There are also some other articles on the use of electron microscopy in legal medicine that are more application-oriented (74/47, 74/197, 74/217). The first articles on the use of CT (80/227) and MRI (102/185) in post-mortem imaging were published. Further studies concern the mechanical stress tolerance of tissues and organs (68/207, 70/178, 74/55, 76/37, 82/305, 102/535).

In the field of *thanatology*, publications on vibices (69/263) and post-mortem haemolysis (81/19) appeared. Studies on the early signs of death are dealt with in 2 articles on livor mortis (69/70, 93/283) and 9 articles on rigor mortis. The publications on late signs of death deal with putrefaction (75/201, 76/293), adipocere formation (72/194, 94/273) and mummification (75/255, 96/279).

43 articles were published on the *estimation of the time of death*. Most of the articles, and the most significant ones, were written by Claus Henssge’s working group. These include systematic studies of the rectal and brain temperature in dependence of the time of death (83/49, 93/1, 93/123) and of the cooling behaviour of bodies recovered from water (92/255). Of great practical importance was the publication of time-of-death temperature nomograms, which made the complicated and time-consuming calculations required until then unnecessary (87/147). By including additional criteria, such as livor mortis, rigor mortis, mechanical and electrical excitability of the skeletal muscles and pharmacological stimulation of the iris, the nomogram based on the rectal temperature was developed into a complex method (95/185). Studies by other research groups determined the levels of potassium, ammonia and magnesium in vitreous humour (71/281, 80/259, 97/259, 102/179). The composition and digestive state of the stomach contents in relation to the time of death were investigated (82/129). Studies on the electrical stimulation of the musculature in the early post-mortem interval were published (85/5, 93/165, 103/435). Reh et al. presented a method for estimating the time of death in bodies recovered from water based on the formation of washerwoman’s skin on hands and feet, autolysis and putrefaction in relation to the water temperature (79/261). Finally, the first entomological studies on the estimation of the time of death in the late post-mortem interval appeared (89/197, 90/303, 91/61, 91/295, 92/39).

Various forms of embolism were described as general *vital reactions*. For example, there are papers on air embolism (92/127, 95/197), fat embolism (69/197, 83/291, 94/309, 99/103) and fibrocartilaginous embolism (72/161). A publication by Pedal et al. on findings obtained by gas analysis to distinguish between air and putrefactive gas deserves special mention (99/151). Other common vital reactions discussed were aspiration (68/195, 68/204, 69/41, 73/273) and exsanguination (74/245, 80/9, 84/99). Numerous papers deal with the proof of vitality using biochemical, enzyme histochemical and immunohistochemical methods. Other articles discuss wound age estimation of skin injuries (70/121), injuries of internal organs (71/1) and brain contusions (84/79).

### Forensic traumatology and pathology

Table 2 gives an overview of the number of publications on all types of violent death.

**Table 2:** Articles on violent death published from 1970 to 1990

Violent death	Number of publications
Blunt force	42
Sharp force	21
Asphyxiation	42
Death in the water	26
Gunshots	40
Electricity	8
Heat	16
Cold	14
Total	209

24 articles on the impact of *blunt force* deal with craniocerebral trauma. A 68-page review paper by Unterharnscheidt on brain damage after craniocerebral trauma deserves special mention (71/153). Articles by Weber regarding post-mortem tests on the fragility of infant skulls (92/87, 94/93, 98/81) were widely discussed. Two letters to the editor debate the findings published by Weber (103/311, 103/313). Blunt abdominal trauma is addressed in 3 papers (80/167, 84/155, 93/143). Specific sequelae following blunt force trauma are presented in papers on subarachnoid haemorrhages (86/149, 87/1, 87/19), skull base ring fractures (67/324, 90/137), skull base hole fractures (93/49), atlas fractures (90/247) and axis fractures (82/89).

10 articles on *sharp force* concern stab wounds. Cut injuries are presented in 2 case reports (72/73, 91/159). Examples of the impact of *semi-sharp* force include injuries from circular saws (85/107, 89/173) and chainsaws (92/215), impalement injuries (91/83) and bite marks (79/73).

Of the articles on *asphyxiation*, 34 deal with strangulation. In 12 papers, possible signs of vitality were examined. These include histomorphological findings in the lungs (81/133, 86/175), Simon’s bleedings (83/283), soft tissue haemorrhages in the larynx (94/127, 99/35), phospholipid concentration in sinus and heart blood (85/29) and thyroglobulin content in heart blood (103/361). Prokop and Wabnitz reported data on the frequency of petechial conjunctival haemorrhages of various origin (67/249). Reh and Haarhoff examined under which hypostatic conditions petechial haemorrhages occur on the head and neck of human bodies (77/47). In 3 studies, fractures of the cervical spine were examined in hanged persons (80/329, 81/299, 82/55). Two articles concern the special form of forearm chokeholds (100/165, 103/309). Two publications deal with the closing of the airways by sand (94/173, 94/191) and one paper with asphyxiation under a plastic bag (70/184). Perthes’ pressure congestion is described in a review paper (81/79).

Most articles on *death in water* concern drowning. In an autopsy study, Haarhoff and Weiler showed that temporal bone haemorrhages are non-specific and therefore not a vital sign of drowning (69/62). Reh discusses the evidential value of the lattice fibre texture of the lung as a sign of drowning (77/219). Three publications are devoted to diatoms (83/319, 85/315, 101/87). One article discusses plant elements in the blood as a sign of drowning (77/219). Five papers deal with the phenomenon of washerwoman’s skin. In two animal experiments, characteristics are described to distinguish drowning in freshwater and saltwater (90/1, 91/47). Possible causes of sudden death in water are discussed in 3 papers (69/1, 80/249, 83/121). Diving accidents are the subject of 3 articles (68/225, 81/157, 95/105).

15 articles on *gunshot injuries* deal with different aspects of shots to the head. They cover signs of close-range shots (88/103, 102/545), the perforation size of cranial defects (73/35), the ability to act (73/61, 76/307), priority in multiple shots (97/213, 98/281, 98/282), and cranial shots in wearers of military protective helmets (102/41). Schneider and Pietrzak discuss bleeding into the shoulder joint space in fatal shots to the head as a possible sign of suicide (95/259). Characteristic features of bullet entrance wounds in the palmar and plantar region were described by Pollak (86/41). One paper deals with gunshot wounds to the liver and spleen (90/167). Wound ballistic characteristics of different ammunition types are presented in 6 papers (73/103, 77/201, 101/219, 103/69, 103/70, 103/137). Two articles describe injuries caused by air guns (84/209, 96/119). Injuries due to blank guns are the subject of 2 publications (75/71, 78/91). Four papers deal with injuries caused by captive bolt guns (68/27, 80/135, 87/279, 90/53). Feensta presented explosion injuries on the basis of the report of 2 cases (96/27).

Three publications on *electrical fatalities* deal with the electric mark (67/293, 85/97, 86, 245). A case report describes the unusual suicide of a student who killed himself by biting into a live appliance cord and simultaneously touching a properly earthed metal fan heater (71/68). In another case report, interstitial microhaemorrhages and leukocytic cell reactions in the muscles beneath the electric marks after exposure to the effects of an electric current survived for several hours are discussed as signs of vitality (87/305). Keil et al. investigated the effect of electricity on the myoglobin content of human cardiac and skeletal muscle (91/185). Bonte et al. discuss problems in the assessment of electrical fatalities in the bathtub (97/7). Wollenek et al. point out that pale streaks on the skin parallel to the water surface in victims found dead in the bathtub are not necessarily electric marks (102/473).

Three autopsy studies were published on deaths due to *heat* (94/71, 95/51, 95/59). In animal studies, the fibrin and histamine content in various organs was investigated (71/114, 72/203, 72/213, 73/149). Maxeiner discusses congestion haemorrhages in the head and neck area of fire victims as vital signs of heat exposure (101/61). Two publications deal with heat haematoma (75/21, 103/227). Schwerd and Müller describe heat damage to hair (100/73). Endris and Beersche investigated heat-induced colour changes in teeth (94/109). In a case report, Staak et al. discuss heat shock as a possible cause of death (72/319). With regard to exposure to moist heat, there is only one publication dealing with vital signs of scalding (83/1).

11 animal studies were published on deaths due to *cold*. Schneider and Klug described increased acetone levels in human urine as a possible indicator of hypothermia (86/59). Madea and Oehmichen discussed haemorrhages and necrosis of the pancreas and haemorrhagic infarction of the descending colon as possible consequences of hypothermia (102/59). Sigrist et al. regarded haemorrhages and microscopic necrosis of the body’s core muscles as “relatively specific signs of hypothermia” (103/463).

Seven of the articles published on *forensic obstetrics* relate to proof of live birth. The umbilical ring (67/119, 67/372), the Wreden-Wendt tympanic cavity test (69/173), the morphology of elastic fibres of the lungs (67/177) and the inflation of the lungs with air (83/303) were examined. Another paper deals with the determination of the body length and age of foetuses based on the size measurements of the ossicles (71/264).

A literature study on *abortion* in Sweden analysed the methods used for this purpose in the period 1870–1970 (70/197). One case report describes an abortion by gunshot (71/108).

Only one publication is available on *filicide*. Walther and Weiler concluded from the results of an autopsy study conducted at the Institute for Legal Medicine in Mainz that the frequency of the killing of newborns as well as the manner in which the offence was committed had not changed between 1948 and 1968.

Five articles concern possible *medical malpractice* in diagnostic procedures. These include the administration of contrast medium (74/207, 76/141, 86/71), a liver biopsy (82/225) and a myelography (75/219). The most frequently described anaesthetic accident is malignant hyperthermia (76/131, 80/117, 94/289). Another fatal anaesthetic accident involves the administration of the anaesthetic gas halothane (72/283). Four papers discuss deaths related to ventilation (71/79, 81/45, 82/289, 94/41). Fatal air embolism occurred after intravenous infusion, bloodletting with a vacuum bottle and arthroscopy (82/211, 85/247, 99/65). Two papers deal with fatal complications of cardiac surgery (83/87, 101/131). Four papers describe resuscitation injuries (86/161, 88/305, 92/67, 98/19). One review paper summarises histological findings in iatrogenic damage (92/1).

Among the articles on *sudden deaths from a natural cause* in adults, papers on sudden cardiac death were the most dominant. Four publications discuss spontaneous aortic ruptures (68/101, 73/53, 79/159, 96/67). In a Swedish autopsy study (101/115), a previously unknown tumour was found to be the cause of death in 41 of 7020 cases. The most frequent causes of death in these cases were tumour metastases, intracranial tumours, pulmonary embolism, haemoptysis and blood aspiration. Other reports on tumour diseases include mesothelioma (76/123), reticulum cell sarcoma (81/329), laryngeal carcinoma (87/129), gastric carcinoma (97/219) and malignant testicular tumours (103/529). Infection-related deaths were Waterhouse-Friedrichsen syndrome (80/73, 88/173), AIDS (95/113), influenza (96/303, 97/165) and tetanus (92/231). Fatal parasitoses were described in 4 articles (69/210, 89/65, 96/235, 98/125). Four publications were devoted to fatal secondary diseases of chronic alcohol abuse (82/113, 89/125, 94/61, 102/231). Three articles dealt with sudden deaths due to allergies (96/79, 102/297, 103/407). There are 24 publications on Sudden Infant Death Syndrome. In most cases, histological examinations of the lungs, the thyroid, the thymus or other organs were performed. Clinical chemistry parameters were determined in 3 studies (70/36, 78/57, 96/277). Two papers dealt with the detection of CMV infections (99/281, 100/237). Hebold et al. performed morphometric measurements of the vertebral arteries (97/41).

### Forensic toxicology

67 articles were published on the *clinical signs of poisoning*. This group contains articles on the symptomatology of poisoning, partly also describing relevant autopsy findings and the results of toxicological/chemical analyses. Special mention should be made of the work of Harzer on fatal poisoning with colchicine after i. v. injection (93/181), of Aderjan and Joachim on fatal poisoning with ethylene glycol (100/199) and of Petkovits et al. on propanolol-2 intoxications (102/69).

24 articles present *pathological anatomical findings* in cells, organs and tissues for the diagnosis of poisoning. Important papers concern the detection of cell changes in smokers (91/37, 95/167), pathological anatomical changes in drug consumption (80/299, 80/305, 93/227) and the problem of toxicological findings in decomposed corpses (93/151).

*Analytical methods* in use during the period under review with various modifications were high-pressure liquid chromatography (97/285, 98/263) and gas chromatography (80/197, 82/43, 99/151), radioimmunoassay (75/275) and enzyme immunoassay (94/219). Atomic absorption spectrophotometry (73/29, 90/105), Fourier transformation infrared spectrometry (95/67) and electron spin resonance (103/599) were also used. Methodologically, the work of Bäumler and Brandenberger on the use of combined spectroscopy (76/159) and of Daldrup on the detection of the designer drug etryptamine with gas chromatography-mass spectrometry, high-pressure liquid chromatography and thin layer chromatography (97/61) deserves special mention. The spectrum of examinations was significantly expanded by the discovery of new pharmaceutical substances (94/197, 100/19) as well as narcotic drugs and doping agents (71/274, 73/219, 79/149, 90/105). Proficiency tests, for example for the detection of thallium, were carried out for external quality assurance of analytical procedures (93/219). The introduction of computers into forensic toxicology is dealt with in the publications by Besserer et al. (78/273) and Maier and Derksen (92/159) as well as Schmidt and Klug (98/249). Machata’s work on calculating retention indices with the pocket calculator (79/193) should also be seen in this context. New analytical materials were used, especially hair and sweat (actually skin excretion products). Thus, methods for the detection of metals (80/79), morphine (84/189), cocaine (98/235), methamphetamine and amphetamine (102/293) as well as methadone in hair have been described. Morphine and methadone (82/251, 103/323) could be demonstrated in sweat.

Of the publications on *alcohology*, 32 articles are concerned with alcohol metabolism and 6 with alcohol analysis. Of particular practical relevance is the work on new alcohol formation in corpses (101/21) and on the genetically determined variability of alcohol metabolism (103/169). Important work on blood alcohol determination by gas chromatography was done by Bonnichsen et al. (71/134) and Machata (75/229). Factors influencing breath alcohol measurements were investigated by Dubowski (76/93).

### Identification of unknown dead bodies

19 articles concerned osteological identification methods. These were studies on sex diagnosis (67/170, 67/175, 69/161, 69/168, 72/255, 75/51, 86/79, 90/199, 90/219, 94/21), age estimation (67/175, 72/40, 84/7, 86/29, 97/223, 103/351), body height estimation (68/149, 74/117, 90/199) and analysis of ancestry (75/51). The determination of the degree of racemization of aspartic acid in dentin was presented as a biochemical method for age estimation with a high estimation accuracy (103/457). Other articles dealt with the estimation of the post-mortem interval of bones (87/275, 91/71). The examination of cremation ashes is covered in 2 papers (77/191, 97/227). There were 3 articles on superimposition (80/183, 80/189, 102/451) and 1 paper on facial reconstruction (89/119). In addition, identification using dentition data was discussed (76/211).

### Forensic genetics

The main focus of the articles on forensic genetics during the period under review was *blood group science*. 20 papers covered erythrocyte membrane systems. 46 papers dealt with serum proteins. Special mention should be made of a paper on the polymorphism of complement factor 6 by Rittner et al. (83/17) and the description of a method for subtyping haptoglobin by Patzelt and Schröder that is suitable for forensic practice (94/207). With 61 articles, the majority of publications were on enzyme groups. A review article by Brinkmann on red cell enzyme polymorphisms in forensic serology (69/83) and a paper on the determination of PGM_1_ subtypes by agarose gel isoelectrofocusing by Weidinger and Schwarzfischer (84/221) are worth mentioning. Three papers (74/121, 75/81, 87/267) were devoted to the HLA system, with which conventional, i.e. gene product-linked, blood group science reached its peak in terms of predictive reliability.

With 58 articles, most of the papers on *trace analysis* are concerned with blood. Three papers deal with blood detection. Seven papers address the determination of species specificity. 46 publications discuss various possibilities of individualising blood traces. Of these, two publications are about sex diagnosis. 29 articles are devoted to blood groups used for trace analysis. Publications on other physiological group properties relate to foetal haemoglobin and alpha-fetoprotein (74/81, 79/79, 79/81, 102/437) as well as pregnancy-specific proteins (69/65, 95/255, 101/9, 102/353). Finally, pathological group characteristics were also described. Studies on the detection of syphilis (91/247) or HIV antibodies (102/487) in blood traces are worth mentioning. 19 articles refer to the examination of semen traces. 13 of them deal with semen detection and 6 with the determination of blood group characteristics in semen. Other publications on trace analysis deal with tissues and organs (20 articles), hair (11 articles), saliva and other body fluids (8 articles), bones (4 articles), teeth (2 articles) and finger and toe nails (1 article).

The largest number of articles on *parentage testing* (35 articles) related to work on calculating the paternity probability of blood group reports (serological reports) using various statistical methods, with some articles announcing the entry of computers into the calculation of paternity probability as well (78/227, 78/243). One paper deals with the calculation of maternity probability in blood group assessment (68/53). Only very few articles were still published on the evidential value of expert opinions on fertility (74/141) and chromosomes (74/17, 84/179, 99/249). In addition, special phenomena relating to blood group serology, such as unequal crossing-over with the risk of misinterpretation, were pointed out (90/27). Furthermore, there are some case reports, for example a twin case with probable superfetation (84/319).

At the end of the period under review, the first publications on *DNA analysis* appeared. First, a paper on sex diagnosis was published (97/21). Studies on the use of DNA polymorphisms in parentage testing and for identification purposes followed (99/241, 102/236, 102/323, 103/235, 103/478). Moreover, studies on DNA stability (103/191, 103/397) were published.

### Scientific and technical criminalistics

Six papers on ballistics were published, dealing with the determination of the firing distance (74/9, 77/121), bullet deflection (69/217, 78/149, 98/241) and problems in the assessment of shots through glass panes (74/167). Two articles are about the examination of saw marks (72/180, 74/145). One publication deals with the detection of tracks, marks or patterns (71/120). Reimann reports experiences with a modified method for demonstration of fibres according to Frei (70/210). Several papers deal with the examination of textile fibres (77/223, 82/237, 96/183) and the transfer of traces from vehicles to clothing in traffic accidents (91/21, 92/291, 94/213).

### Clinical legal medicine

In a review paper, Naeve and Lohmann (72/79) discuss the “Methodology and legal relevance of immediate physical examination of living persons after criminal offences” on the basis of cases from the Hamburg Institute for Forensic Medicine and Criminalistics. As a reason for the fact that in West Germany medicolegal experts were relatively seldom entrusted with the clinical examination of living persons, the authors point to the consequences of the “Law for the Unification of the Public Health System” of 1934, on the basis of which some medicolegal tasks were transferred to the public health offices. As a result of their considerations, the authors conclude that the examination of victims of violence and accused persons belongs into the field of legal medicine, since knowledge and experience in legal medicine are required for the proper collection and assessment of findings usable in court.

### Forensic psychiatry and psychology

Two papers report on chromosome analyses in mentally ill offenders (68/138, 69/157). Other publications deal with the psychopathology of hashish abuse (68/57), exorcism (82/313) and the so-called collective-crime murder of a lover (95/137).

### Sexual medicine

In one article, the phenomenon of female exhibitionism is described and compared with the characteristics of male exhibitionism on the basis of personal observations and a literature review (71/126).

### Traffic medicine

Most of the publications in traffic medicine concerned the examination of traffic accidents under medicolegal aspects. Special accident constellations, such as pedestrian-car accidents (67/282) and accidents involving a two-wheeled vehicle (101/1), were presented. A study examined the importance of crash helmets worn on the survival chances of riders and passengers of motorised two-wheelers (75/235). Specific injury patterns, such as neck injuries (72/2), aortic ruptures (74/235), the wedge-shaped Messerer fracture (75/163) and decollement (85/211), were described. A review paper dealt with the medicolegal reconstruction of aviation accidents (71/251). A deceleration sled system for performing traumatomechanical investigations on corpses was presented (74/25). Finally, traumatomechanical experiments on airbag and seatbelt injuries were published (72/8, 72/22, 74/31). There were 8 toxicological studies on the influence of alcohol (76/187), hashish (73/131) and pharmaceutical drugs on the fitness to drive (67/19, 79/225, 80/319, 93/95). Also discussed were possible physical limitations that may affect driving ability, such as heart disease (81/1), pacemaker implantation (67/42, 72/32) and Wernicke’s encephalopathy (80/225).

### Social medicine

One publication examines the circumstances of death of opiate addicts in outpatient care (96/215). Another paper discusses social medical aspects of fatal cancers (100/259).

### Criminology

Most articles concern alcohol and drug delinquency (70/25, 82/1, 83/209, 92/169, 97/141, 102/509, 103/63). Other papers analyse homicide situations in loose partnerships (71/39) and defensive body dismemberments (81/151).

## Discussion

### General

The *Deutsche Zeitschrift für die gesamte gerichtliche Medizin*, founded in 1922, was continued from 1970 to 1990 under the new title *Zeitschrift für Rechtsmedizin—Journal of Legal Medicine*. The internationalisation intended with the name change of the journal was successfully achieved. Of the 1416 articles published in the period under review, 411 were written by authors from non-German-speaking countries. 380 articles were in English. In retrospect, the 1970–1990 phase in the journal’s 100-year history thus represented an intermediate stage between the German-language period of 1922–1969 and the English-language period beginning with the journal’s 104th volume.

In addition to 990 original papers, 323 case reports, 72 reviews, 18 letters to the editor, 10 obituaries and 3 editorials were published. The relatively high proportion of case reports in the total number of scientific articles (23 percent) is remarkable. Madea et al. [10] pointed out the special importance of case reports for legal medicine, which is also due to structural features of the discipline. For example, medicolegal research is often observational research as opposed to the experimental research that predominates in clinical trials. Medicolegal research can largely only be observational research, since relevant facts, such as violent bodily harm, violation of sexual self-determination or the effect of alcohol and drugs on driving ability and criminal responsibility, can often not be simulated for ethical reasons. Moreover, individual case reports often act as pacemakers for generating hypotheses or for carrying out systematic investigations.

Table 3 compares the percentage shares of the topics covered in the three periods under review so far. In the period 1970–1990, the number of papers on forensic genetics increased significantly, and there was a small increase in publications on the identification of unknown dead bodies. An opposite trend was observed in the articles on forensic psychiatry and psychology, sexual medicine and social medicine. This development reflects a further sharpening of the discipline’s profile.

**Table 3:** Comparison of the percentage share of the topics in the three periods under review

Topic	1922–1944	1948–1969	1970–1990
History and development of the discipline	1.6	0.5	0.6
Personalia	1.9	2.7	0.7
Legal issues and expert activity	7.9	3.9	3.7
General legal medicine	10.3	9.6	14.4
Forensic traumatology and pathology	32.2	28.2	27.8
Forensic toxicology	16.6	25.3	19.6
Identification of unknown decedents	1.2	2.1	2.7
Forensic genetics	8.5	15.9	23.5
Scientific-technical criminalistics	6.4	1.2	1.7
Clinical legal medicine	0.4	0.6	0.1
Forensic psychiatry and psychology	4.3	2.6	0.6
Sexual medicine	3.2	1.4	0.1
Traffic medicine	1.1	4.2	3.1
Social medicine	0.6	0.6	0.1
Criminology	3.8	1.2	1.3
Total	100	100	100

Without claiming to be representative or even complete, some of the key areas of medicolegal research in the period under review will be discussed below.

### History and development of the discipline

Significant articles on the history of legal medicine were not published. However, there are three papers on the conception and development of the discipline, in which thoughts were expressed that are still relevant today. Two papers deal with the role of legal medicine in ethics committees (92/247) and the significance of morphological, especially histological, examinations (100/5). However, the article “Forensic Medicine and Criminalistics” (102/421) by Schwerd deserves special emphasis. The article is not entirely without historical considerations. The starting point of Schwerd’s reflections is the question of how the existential crisis of the discipline could have come about, which induced the German Science Council in 1966 to recommend dissolving the discipline of legal medicine and assigning the individual fields to other disciplines. Schwerd sees the cause of this crisis in the disunity of the discipline in the first half of the twentieth century, which in particular lacked a clear demarcation of legal medicine from social medicine and criminalistics. This disunity was expressed by 5 different names for the institutes in Germany: Institute of Legal Medicine, Institute of Legal and Social Medicine, Institute of Legal Medicine and Criminalistics, Institute of Legal Medicine and Scientific-Technical Criminalistics and Institute of Legal Medicine and Insurance Medicine. Schwerd sees a return to the two core areas of legal medicine as a way out of the crisis. The first core area, he said, was the inspection of the scene of death, followed by the autopsy. In particular, in cases of homicide, Schwerd considers the inspection of the scene of death to be indispensable. Because, if the medicolegal expert doing the autopsy does not have a detailed knowledge of the circumstances of the case at the beginning of the autopsy, let alone has been able to form his own impression of the situation, then a useful result becomes a matter of chance. In this context, Schwerd objects to the autopsy of the “naked body”, in which he sees the concept of “forensic pathology” which originally came from the Anglo-American sphere. Moreover, he rejects the term “forensic pathology” for three reasons. First, violent death is not a disease. Second, the term suggests that legal medicine is a branch of pathology, which it has never been. Third, the term “forensic pathology” has often become synonymous with superficiality and should therefore be rejected by medicolegal experts, who should apply special thoroughness. As the second core area of legal medicine, Schwerd identifies medical trace analysis, which he understands to mean the examination of medical evidence using special methods, including histological and toxicological examinations.

### Medicolegal methods

According to Schwerd, legal medicine is a medical discipline that involves the application of medical knowledge and methods in teaching, research and practice for juridical purposes (102/421). In medicolegal research, new methods are often adopted from other disciplines and tested for forensic applications. However, this adoption does not always prove successful. For example, electron microscopy was tested for various forensic issues during the period under review. Thrombi (74/47, 75/217), cut wounds (72/11, 74/197), electric marks (67/293), gunshot wounds in bones (73/137) and burnt bones (77/191) were examined under the electron microscope, but it was not possible to establish the use of electron microscopy in medicolegal practice. According to Adebahr, this is because electron microscopic examinations are suitable for recognising normal structures, but not for assessing structural abnormalities. Because in the orders of magnitude achieved by these investigations, an abnormality or damage appears almost uniform (103/477). Another reason is the artefacts of ultrastructural formations caused by autolysis and putrefaction, which can lead to problems in the evaluation of examination material taken during forensic autopsies (92/77). More successful was the use of new radiological methods for post-mortem imaging. The first publications on CT (80/227) and MRI scans (102/185) of corpses appeared during the period under review. These papers can be seen as pioneers of virtopsy, which has experienced its breakthrough in the next period under review [19].

### Time of death

In the field of general legal medicine, works on thanatology and estimating the time of death were also published. A significant research contribution to the estimation of the time of death in the early post-mortem interval was made by the working group led by Claus Henssge. As early as 1962, Marshall and Hoare [11] had published a two-exponential model for body cooling. However, the feedback was extremely low [6]. The breakthrough of rectal temperature-based time-of-death estimation in forensic practice only came after Henssge’s investigations to improve this model, which he conducted at the Institute of Forensic Medicine of the Charité in Berlin (83/49). An essential contribution to practicability was made by the publication of nomograms for reading the time of death, which replaced complicated and time-consuming calculations (87/147). A multicentre study confirmed the suitability of the nomogram method in practical use (103/257). The range of applications of this method was extended by studies on bodies stored in water (92/255). Further studies concerned systematic measurements of central brain temperature by Brinkmann et al. (78/69, 81/207) and Henssge et al. (93/1) and the combined measurement of brain and rectal temperature (93/123). In 1984, Henssge et al. published a complex method in which, beside the nomogram method, livor mortis, rigor mortis, and the mechanical and electrical excitability of skeletal muscles as well as the pharmacological stimulation of the iris were taken into account as additional criteria (95/185). The Henssge nomograms have meanwhile been included in most of the world’s textbooks [6]. With the nomogram method, time-of-death estimation has been raised to a higher quality level [20]. In a recent review, Potente and Wirth [15] assume that the Henssge method will continue to make important contributions to criminal investigations of deaths in the years to come.

### Forensic toxicology

The challenges of forensic toxicology at the beginning of the 1970s were the multitude of new pharmaceutical drugs, a change in analytical methods and a lack of substance-specific measurement parameters [14]. Among the newly approved pharmaceutical drugs were the benzodiazepines, the detection of which, according to Daldrup and Klug [1], marked the “beginning of modern forensic toxicology”. The chemist Leo Sternbach had synthesised chlordiazepoxide, the first benzodiazepine approved as a tranquilliser, in Basel in 1958. Shortly afterwards, the synthesis of the now widely used diazepam followed. Benzodiazepines represented a special challenge for forensic toxicology, as the low amounts of active substances could no longer be detected with conventional analytical methods and as the formation of active metabolites made their detection absolutely necessary [1]. In particular, Harald Schütz from the Institute of Legal Medicine in Giessen did intensive and systematic work on the detection of benzodiazepines. An example is the paper “Gaschromatographic data of 19 derivatives of hydrolysis from 12 important benzodiazepines and 17 main metabolites” (82/43), published in 1978.

Another research focus in forensic toxicology was the development of alternative examination materials such as hair or sweat. The examination of hair to detect the consumption of narcotic drugs, but also of other pharmaceutical substances, alkaloids and even alcohol, plays an essential role in today’s forensic toxicology [1]. One of the first papers to detect morphine in hair was published by Klug in 1980 (84/189). The detection of drugs and medicines in sweat is also significant for the practice of forensic toxicology, as the active participation of the person to be examined is not necessary for the collection of the sample. As early as 1979, Ishiyama et al. published a paper on the detection of morphine and methamphetamine in sweat (82/251).

Pioneering activities for external quality control of forensic toxicological analyses were the organisation of proficiency tests [14]. The results of the first proficiency test for barbiturate determination were published by Machata [9] in 1970. A proficiency test for the determination of thallium in urine was conducted by Geldmacher-von Mallinckrodt et al. (93/219). Since 1995, the Society of Toxicological and Forensic Chemistry (GTFCh) has been organising proficiency tests relating to forensic and clinical toxicology as well as to therapeutic drug monitoring in cooperation with ARVECON GmbH. In 2017, 76 proficiency tests were conducted [17].

### Forensic genetics

In the field of forensic genetics, the serological research already reported in the first two parts of the article series was continued in the period under review and reached its quantitative peak. New research results were presented on the erythrocyte membrane systems, on serum proteins and on enzyme groups, which accounted for the largest share in terms of quantity. Since the discovery of the first trait of a genetic polymorphism on leukocytes by Dausset [2], the HLA traits had evolved into the most polymorphic conventional blood group system. Three papers on the HLA system were published in the *Zeitschrift für Rechtsmedizin—Journal of Legal Medicine* (74/121, 75/81, 87/267). The diagnostic possibilities of blood tests on biological traces have been expanded to include the detection of foetal blood as well as physiological and pathological group characteristics. Today, however, serological papers on trace analysis and parentage testing are mainly of historical interest. In 1985, the discovery of genetic fingerprinting by Alec Jeffreys ushered in a new era in forensic genetics [4]. At the end of the period under review, the first articles on genetic fingerprinting also appeared in the *Zeitschrift für Rechtsmedizin—Journal of Legal Medicine* (99/241, 102/236, 102/323, 103/235, 103/478).

### Traffic medicine

**Fig. 3:**
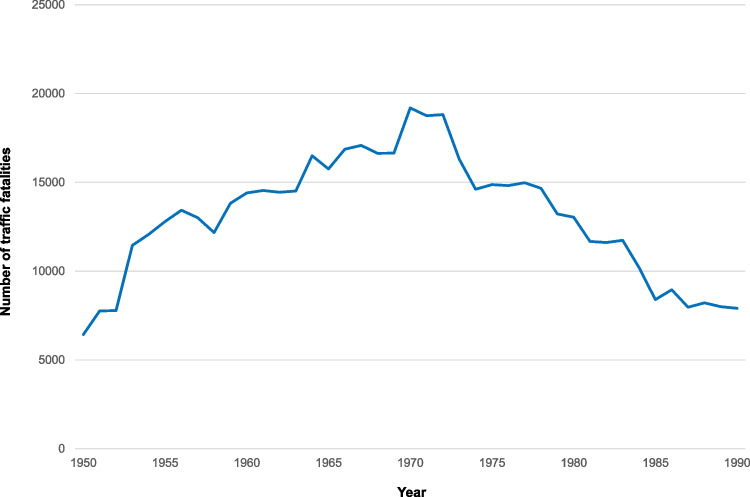
Number of traffic fatalities in West Germany from 1950 to 1990. Source of data: Statistisches Bundesamt [18]

The preventive function of medicolegal research was perhaps most evident in the field of traffic medicine during the period under review. In view of the sharp rise in the number of traffic accident fatalities, which reached its maximum in 1970 (see Fig. 3), medicolegal research on road accidents was intensified in order to increase vehicle safety. Traumatomechanics was established as a new field of research at the Heidelberg Institute for Legal Medicine and Traffic Medicine [13]. Traumatomechanics deals with the study of the quantitative relationship between mechanical stress and injuries to the human body. For traumatomechanical experiments, a deceleration sled system was installed at the Heidelberg Institute (74/25). The results of the investigations on corpses protected by seat belts accelerated the introduction of the obligation to wear seat belts in Germany and contributed to the optimisation of seat belts and airbags (72/8, 72/22, 74/31). Child restraint devices, side impact protection, safety helmets and crash barriers have also been improved through research into the traumatomechanical properties of the human body [12]. By 1990, the number of traffic accident fatalities in West Germany was more than halved in spite of an increasing volume of traffic (see Fig. 3). Traumatomechanical research achievements played a significant role in this result. It is estimated that, based on all studies on corpses worldwide, 60 lives have been saved per investigated corpse in the US alone, and a multitude of injuries have been mitigated or prevented [7].

The prevention of road accidents also involved fundamental research into the assessment of the fitness to drive, which can be impaired by alcohol, other intoxicants, medication or physical deficiencies. Toxicological research in Germany and Switzerland benefited from the locational advantage that it was legally possible to take a blood sample if there was reasonable initial suspicion of drunken driving without the consent of the person concerned [1]. In extensive series of tests on these blood samples, for example, the influence of benzodiazepines and other pharmaceuticals on road users has been demonstrated (80/319, 93/95). These investigations had only become possible because new procedures allowed the processing of even small amounts of residual blood from blood alcohol determinations [8].

On the occasion of the 100th anniversary of the German Society of Legal Medicine, Eisenmenger [3] paid tribute to the achievements of German-language research in traffic medicine as follows: “All legal regulations on the question of the fitness to drive under the influence of alcohol and drugs, but also the compulsory use of seat belts and helmets, would be inconceivable without scientific research of legal medicine.”

## Conclusions

The articles published in the *Zeitschrift für Rechtsmedizin—Journal of Legal Medicine* between 1970 and 1990 show how the main research areas in legal medicine developed through the interaction between the requirements of the administration of justice and the technological possibilities. While electron microscopic examinations could not establish themselves in medicolegal practice, first publications appeared on the use of CT and MRI in post-mortem imaging, which became very important in the following decades. A large number of original papers and case reports have expanded the body of knowledge on the diagnosis of forensically relevant forms of violence. These findings were of benefit to the administration of justice and thus to legal certainty. The analytical methods commonly used in forensic toxicology at the beginning of the 1970s were improved and supplemented by new methods. Not only was it possible to significantly reduce the detection limits of pharmaceuticals and drugs but also to examine new analytical materials. The relative proportion of serological papers grew in comparison to the periods under review in the first two parts of our article series and reached its maximum. With the introduction of the DNA analysis at the end of the 1980s, however, the serological techniques lost all practical significance. When the number of traffic fatalities reached a peak in 1970, traumatomechanical research to increase vehicle safety and basic toxicological research to improve the assessment of the fitness to drive were intensified. The results of these efforts contributed significantly to a reduction in road accident fatalities.

## Footnotes

1 The first number refers to the volume, the second to the start page.

2 This orbituary is not included in the print version of the journal.
